# *Comparative Neuropathology* (1962): Attending to Neuropathologies Across Multiple Species

**DOI:** 10.1093/jhmas/jrz014

**Published:** 2019-03-19

**Authors:** Anna Kathryn Schoefert

**Affiliations:** King’s College London, Department of History

**Keywords:** Neuropathology, Veterinary medicine, Comparative medicine, Neuroscience, Animals in medicine

## Abstract

This paper takes as its subject *Comparative Neuropathology* (1962), arguing that the volume illustrates the interlocking cultures of veterinary medicine, human medicine, and laboratory-based biological sciences after the Second World War. The project amassed cases of domestic, experimental, and wild animals, identified species-specific conditions, and evaluated the vulnerabilities of the nervous system to disease and trauma. The collection of ill ruminants, poisoned cats, and injured dogs built on earlier traditions of comparative medicine, but also reflected the turn to biological principles to explain medical conditions, increased industry and military funding for the biomedical sciences, and changes in veterinary practice. Using *Comparative Neuropathology* as a lens, this paper probes the actors, affiliations, and frameworks that wrestled with new species of neurological patients, newly exposed vulnerabilities of the nervous system, and the emergence of new neurological sciences, casting new light on the heterogenous landscape of the emergent neurosciences and mid-twentieth-century efforts to entwine human and veterinary medicine.

Animal brains were only partially familiar subjects in 1962 when two American-based veterinary pathologists, James Robert Maitland Innes (1903-1974) and Leon Z. Saunders (1919-2009) published the 800-page tome, *Comparative Neuropathology.*^1^ Neuroanatomists and experimentalists had elaborated, compared, and mapped out the brains of primates, dogs, cats, mice, but the species choice and brain maps reflected experimentalists’, not veterinary needs. Knowledge of animal brains was more likely to shape medical neurology than veterinary medicine. Around 1870, dogs had prompted neurophysiologists to correlate brain lesions with loss of function, and a monkey’s gait had spurred the prominent neurologist Jean-Martin Charcot to see animals as models for human patients.^2^ Animals’ species-specific vulnerability to neurological disease, damage, and disorder, however, received only scattered attention until the mid-twentieth century. *Comparative Neuropathology*’s efforts to collate neuropathological cases across species marked a significant shift around 1960 carried equally by developments in experimental research and veterinary medicine.

Its collection of dogs with canine cancers and distemper, pure-bred cattle with congenital brain malformations, foxes transmitting encephalitis, colonies of germ-free mice contaminated by clinically invisible microorganisms, cats poisoned by fish, firstly, reflected widespread interest in the nervous system after the Second World War. The species range, secondly, showcased a growing market for pet services, new specialities dedicated to laboratory animal care, an increased emphasis on veterinary public health in international health campaigns, and an interest in chronic illnesses. *Comparative Neuropathology*’s ill sheep, coughing mice, injured dogs, and unsteady calves, thirdly, indicated complicated alignments of veterinary medicine, human medicine, and the biological sciences. Its authors argued that cases of nervous system disorders and diseases that arose spontaneously provided crucial insights into the vulnerabilities of the nervous system constituting an important corrective to findings established solely in experimental settings or in clinical practice with human patients. Their discussion was to prove that veterinary medicine and veterinary pathology were biomedical research subjects with a reach far beyond the farm, field, home, or zoo.^3^ This paper follows their account and reviews *Comparative Neuropathology* as a corrective lens of the histories of mid-twentieth-century neuroscience and veterinary medicine. In the literature on mid-twentieth-century neurology and the neurosciences to date, animals have featured predominately as experimental subjects in laboratory settings.^4^ Analysing the rise of the neurosciences in the 1960s Joelle Abi-Rached and Nikolas Rose identified the emergence of a characteristic new thought style, the “neuromolecular gaze,” that scrutinised complex phenomena such as consciousness or cognition in terms of molecular biology, neuronal networks, neurochemical transmitters, and cellular structures.^5^ This gaze examined animal brains as a matter of course to model behaviour, disease, neural structures, and neural functions.^6^

*Comparative Neuropathology* treated animals differently even though it was rooted in the structures of experimental research. In its pages, animals were not only experimental subjects and models which were to reveal general truths about the nervous system, but also ill beings, patients, members of herds, economic resources, husbanded subjects. The volume emphasised diversity of species and reactions and stressed the limitations of focusing exclusively on neurological features, ignoring field observations, or disregarding clinical features. In *Comparative Neuropathology* the brain was particular—species-specific and embodied—differing significantly from the assumptions governing many neuroscience experiments. Yet the volume’s composition also exemplified the hybridity of the neurosciences and the hopes for new economic and scientific trading zones for research on brains that Abi-Rached and Rose noted.^7^ However idiosyncratic, as an account of neuropathological conditions known to date *Comparative Neuropathology* provided not only an outline of current understandings of neurological disease entities, but also a snapshot of a heterogenous landscape of the 1960s: the growing number of neuroactive and neurotoxic substances, the shifting forms of veterinary practice including calls for “One Medicine” and alliances with comparative medicine, the emergence of the neurosciences as a disciplinary field, and hopes for strong, cross-disciplinary research platforms.

The analysis follows those historians' troubling monolithic accounts by attending to the retrospectively motley and marginal cases in the brain (and mind) sciences, in particular those turning to a close reading of techniques and technologies in Delia Gavrus and Stephen Casper’s recent edited volume.^8^ Even at the time of publication, *Comparative Neuropathology* was a highly specific, marginal area in neurological studies, yet it provides an extraordinary lens to review the existing accounts on kuru, scrapie, and other mid-twentieth-century neuropathological concerns and expands the scholarship on comparative practices in the mid-twentieth-century life sciences more generally.^9^ This paper draws predominately from published sources to scrutinise Innes and Saunders’s volume as an assemblage joining multiple constituencies, some of them previously underexplored in the histories of neuroscience and histories of veterinary medicine alike.^10^ It points to career trajectories that spanned veterinary schools, collaborations with medical pathologists, pharmaceutical industry, chemical warfare laboratories. It also unearths overlaps with concurrent proposals for a medicine that transcended species boundaries, subsequently labelled “One Medicine.” Whereas this field was, and continues to be, mainly associated with transmittable diseases, the amalgamation of cases in *Comparative Neuropathology* marked a contemporaneous interest in chronic, non-infectious diseases and a network of allies yet unexamined.^11^

The first half of the paper discusses Innes’s efforts to make neuropathologies in animals visible to larger audiences in the 1930s, before situating *Comparative Neuropathology* in its new post-war audiences in military and industrial research laboratories and in private veterinary medicine. The second half of the paper probes the attempt to produce an all-encompassing, coherent comparative neuropathology and positions *Comparative Neuropathology* in a series of campaigns to present a unified cross-disciplinary front around 1960—be it in neurology, veterinary medicine, the neurosciences, or the biological sciences.

## Innes’s case of swayback: Creating audiences for veterinary neuropathology

*Comparative Neuropathology* was published in 1962. Four decades earlier, when Innes began his career as histopathologist and veterinarian in 1920s Britain, veterinarians’ interest in animals’ susceptibility to neurological disorders had been marginal, with the exception of infectious diseases and birth defects.^12^ Summaries of animal neuropathology were more likely to be found in handbooks of human neurology, where authors stated summarily that non-infectious neurological disorders were rare in animals.^13^ Incidence rates, however, were difficult to establish. Veterinarians saw few neurological cases and had few means of evaluating them. The neurologists’ arsenal of reflex tests, X-rays, lumbar punctures, encephalography, and subjective patient accounts were either unavailable, inappropriate for many species, or financially not feasible for practicing veterinarians and their clients. Post mortem neuropathological examinations, a standard tool for confirming neurological diagnoses, were too time-intensive. The scattered publications on nervous system disorders in animals discussed conditions with clear economic repercussions and sizable patient populations: infectious diseases, spinal cord injuries in horses and dogs, or *Dummkoller* and other equine hydrocephalic conditions. By contrast there was little written on sporadic nervous disorders, tumours, non-fatal diseases with neurological components such as degenerative disorders, or systematic neuropathological reviews post mortem—all of which were established research fields in human neurology.^14^

Lack of interest in animals’ susceptibility to nervous disorders stood in contrast to the wealth of studies on animal brains in other settings. Innes’s mentor O. Charnock Bradley, the chair of anatomy at Edinburgh’s Royal Dick Veterinary School, had written his 1905 doctoral dissertation on the morphology and development of the mammalian hind-brain, contributing to a fin-de-siècle groundswell of projects on configurations, cell layers, morphogenesis, and evolutionary trajectories of the mammalian nervous system.^15^ Most studies concentrated on cats, dogs, primates and other species central to neurophysiological research, or focused on evolutionary outliers or representative species. Sheep, cows, pigs, and other species seen in veterinary practice received far less attention in neuroanatomy—and even less in neuropathology.

British veterinarians had argued that knowledge of animal diseases was relevant for human medicine at least since the 1830s.^16^ In the late nineteenth century, collaborations had turned hostile as clinicians, veterinarians, bacteriologists, and public officials battled for professional standing and authority. In 1924, when Innes graduated from veterinary school in Edinburgh, the sides were still entrenched. Some veterinarians—among them Bradley—were beginning to call for closer alliances to investigate spontaneously arising illnesses in “man and lower animals” to identify more general, “biological” principles of pathology.^17^ This new call for a “comparative” medicine resonated with medical practitioners keen to counterbalance increasing specialisation with a generalist outlook. Bradley’s comments also sought to assert veterinarians’ professional authority and aver their relevance for human health. Given the limited institutional resources for veterinary science and widespread underemployment among veterinarians, he feared that medical researchers were flooding into veterinary medicine to chase new public and private funding for distemper and other high-profile infectious diseases in animals.^18^ In 1926 Innes took up a position at one of the new institutions, the Institute of Animal Pathology in Cambridge but throughout the 1930s he also leaned on medical researchers for further training in histopathology.^19^ In retrospect he would attribute his inter-war scientific career to these co-operations which allowed him to circumvent the dominance of bacteriology in animal pathology.

In his papers from the mid-1930s Innes stressed that the closer study of animals was indeed central to fully explaining disease processes in man and animals alike.^20^ In his analysis of swayback, a disease in newborn lambs, Innes set out to demonstrate that veterinary research contributed to neurology, neuroanatomy, and neuropathology. Outbreaks of swayback were limited to a few British counties, but had also been reported in Australia, Peru, and elsewhere where large-scale losses were common. The disease was sporadic, but struck all breeds, could occur year after year, and led to significant economic losses. Clinical symptoms included a severely uncoordinated, staggering gait, paralysed hindlegs, and blindness at birth; affected lambs typically succumbed in days though they could be kept alive by bottle feeding.^21^ The neuropathological picture was dominated by the destruction of myelin, the substance of nerve sheaths of the cerebral white matter. Swayback therefore fell within a large, unwieldly class of demyelinating diseases that preoccupied inter-war neurologists, neuropathologists, and neuroanatomists who disagreed about classifications and causes of demyelinating diseases, puzzling over their inversion of nerve growth and variously proposing links to infections, allergies, hereditary factors, toxins, or environmental conditions. Suffering from a demyelinating syndrome that likely began *in utero*, swayback sheep intrigued, particularly as researchers largely lacked experimental models for demyelinating diseases. In his papers and talks Innes highlighted that veterinarians were uniquely able to study intermediate phases of the disease. Animals suffering swayback could be kept under observation, fed artificially, and killed to arrest the disease at specific stages.^22^ Flocks could be subjected to transmission experiments, tested for nutritional deficiencies, and exposed to varying environmental conditions. Swayback therefore had implications beyond farming and veterinary medicine, particularly as its pathological features resembled Schilder’s encephalitis, a rare fatal condition in human infants. Innes hoped that studying swayback would “[suggest a new approach] towards solving at least the mechanism of demyelination.”^23^

Innes presented his studies to the Comparative Medicine Section at the British Royal Society of Medicine and published his papers in journals of veterinary medicine and human neurology. In the next decade he expanded his discussions to other demyelinating diseases and other neuropathologies, arguing that only a comparative perspective could disclose whether different species showed different reactions of nervous tissue to the same harm, thereby elucidating the range of normal and abnormal reactions of the mammalian brain. Studies of swayback gained new relevance when four out of seven researchers of swayback were struck with neurological disorders in the 1940s, raising questions about interspecies transmission. By then, large-scale field experiments had already linked swayback to copper malabsorption in pregnant ewes—which raised the potential of new treatment regimes for other demyelinating diseases.^24^ Swayback, its classifications, and its analogies among human diseases remained debated. Neuropathology textbooks, published between 1944 and 1962, variously considered swayback among the demyelinating diseases, aging and degenerative processes and nutritional deficiencies.^25^

The case of swayback demonstrated the careful rhetorical links comparative neuropathology drew between medical pathology and veterinary pathology. Innes sought to make comparative neuropathology familiar enough to benefit from funding, prestige, research opportunities and other resources available to medical pathologists, while also insisting on the specificity of veterinary knowledge and its significance in unravelling mechanisms of demyelination, disease classifications, or the range of reaction of the nervous tissue to injury, disease, degenerative processes. In the introduction to *Comparative Neuropathology*, Innes and Saunders similarly distanced veterinary research from animal husbandry and aligned it with other sites of medical research, positioning veterinary specialists as uniquely prepared to identify commonalities and differences. In general, they cautioned against inferring “causal connections” between swayback in sheep and their “pathological counterpart” in a human condition, while emphasising that the disease in lambs highlighted the significance of copper and other trace elements in brain health more generally.^26^ The introduction gestured towards comparing conditions in humans and animals, but the bulk of the text railed against applying disease concepts established in human neurology to veterinary patients “without much supporting pathologic evidence” and against studies exclusively concentrated on disease processes in humans.^27^

## Revealing a world of proliferating neuropathological conditions, 1940-1960s

With roots in inter-war comparative medicine, *Comparative Neuropathology* was shaped by new post-war configurations. It presented a world in which neurological diseases and disorder seemed to be proliferating. The “many ‘new diseases’ … identified in recent years … [might not be] essentially ‘new,’ but … more meticulous study of animals has revealed new facts, … some ‘new’ diseases [may] have been jumbled up as a clinical complex with others.”^28^ Its collection of cases illustrated that the “jumbled” definitions could not account for all new discussions of neurological disorders. Among the new syndromes was Minamata disease first observed in humans and cats of a fishing community in a Japanese cove, Minamata Bay, in 1953. Epidemiologists linked it to fish consumption, which further studies showed to be contaminated by mercury-laden industrial waste.^29^ Others were associated with the neurotoxicity of drugs that attacked sensory nerves. Published the same year as Rachel Carson’s *Silent Spring*, *Comparative Neuropathology* foresaw widespread hazards with “the advent of the newer insecticides” that were “notorious for their high toxicity and sudden death, preceded by muscular spasms, fibrillary twitchings, clonic and tonic spasticity […],” signs of an affected nervous system yet to be fully understood.^30^ The scale of man-made diseases alarmed and provided new audiences for neuropathological expertise in diagnosing nervous tissue, both in the laboratories making new potentially neurotoxic substances and in those evaluating their effects.^31^

Mindful of his own career path, Innes attributed veterinary pathology’s, and veterinary neuropathology’s, post-war professionalisation and growth to American military support.^32^ Though not a member of the armed forces, Innes was employed in military-funded laboratories on both sides of the Atlantic after the Second World War. He had left Cambridge in 1939 to join the new pharmaceutical department at British Imperial Chemical Industries (ICI), frustrated by the lack of prospect of a permanent academic position.^33^ At the brink of the Second World War ICI had been one of several chemical companies to open and then rapidly expand medical chemical sections and biological laboratories with attendant animal breeding colonies, veterinary field stations, and animal testing routines, therefore necessitating veterinary expertise.^34^ In 1948, Innes’s experience at ICI translated into a role setting up a laboratory animal colony at Porton Down, the site of British research into chemical and biological warfare agents. After emigrating to the United States in 1948, first as a special research fellow at the National Institute of Health, in 1952 Innes became the chief of pathology at Edgewood Arsenal in Maryland, Porton Down’s American equivalent and close collaborator. He held the role until 1958, when he moved to Brookhaven’s National Laboratory’s biological section in New York state, where research was supported by the US Atomic Energy Commission, staying until 1963. *Comparative Neuropathology* was written with this institutional support that funded not only Innes’s, and Saunders’s, salaries but also provided technicians, photographers, illustrators, and librarians.^35^ The co-authors’ career tracks in military institutions and private industry resembled each other, though Saunders, a Canadian veterinarian, had served in the armed forces during Second World War and subsequently held positions in the American reserves, assigned to the Armed Forces Institute of Pathology.^36^

Nervous system disorders constituted a key theme for American post-war military researchers studying psychotropic substances, aviation medicine, psychological warfare, and weapon development. In the late 1940s, Edgewood (and Porton Down) were being scaled up to evaluate a new class of highly lethal nerve agents synthesised in Nazi Germany whose existence had come as a surprise to the Allies at the end of the war.^37^ Tobun, Sarin, and others were easy to disperse, effective at low concentrations, difficult to detect, and frighteningly quick and graphic in their action as experiments on animals showed. Neuropathologists were among the researchers scrutinising these substances. An official document, a 1956 personnel inventory, identified a civilian neuropathologist on staff, Innes.^38^ It defined Edgewood as a chemical warfare laboratory, but specified that the site was also researching and developing “offensive and defensive … biological, smoke and flame warfare” agents, the associated munitions systems, and the pilot plants they necessitated. As Edgewood’s chief of pathology Innes directed a team of 22 histopathologists, bacteriologists, biologists, technicians, and veterinary laboratory officers, half of them civilian, who were tasked with “formulat[ing] and conduct[ing] research on effects of chemical warfare agents on animals with particular emphasis on gross and anatomic changes and on infectious aspects” using clinical and pathologic anatomical examinations.” His section scrutinised “insecticides, rodenticides, and other toxic military chemicals” and their effect on animal bodies, commenting on possible therapeutic regimes to counteract or ameliorate the damage these substances caused. The laboratory, moreover, ran training courses in pathology for physicians, veterinarians, and medical pathologists, thereby creating an important interface of veterinary and medical research.

The remit of the pathology branch stretched across multiple organ systems, though Edgewood’s medical laboratory also included a branch dedicated to neurology. Cooperation between branches was said to be close and animals were key to large series of laboratory and field experiments. Animal subjects identified lethal doses, possible routes of exposure, and tissue damage. In field studies, animals such as sheep “keeling over” acted as sentinels for the presence of nerve agents in tests of gas masks and other protective clothing.^39^ Innes’s publications from the period attested to exposure and toxicity studies in sheep, goats, pigs, as well as primates, rabbits, guinea pigs, and mice. Papers commented on concomitant murine pneumonia, species choice in chronic inhalation experiments, common experimental errors and nervous system damage due to irradiation or pesticide, indicative of Innes’s responsibilities in veterinary pathology, toxicology, and animal breeding as well as neuropathology.^40^ Available outside military circles these accounts seldom expanded on the results of toxicity tests, the relative effects of exposure, or the substances being evaluated but they indicated the scale of research on animals and on the vulnerability of the nervous system being pursued under the auspices of the military.^41^

In the immediate aftermath of the Second World War, veterinary pathology had benefitted from the patronage of officers at the Army Medical Museum (later the Armed Forces Institute of Pathology) who were conducting training courses for medical pathologists, setting up a registry of veterinary pathology and consolidating collections with calls to expand its neuropathological holdings. In the late 1940s they supported establishing the American College of Veterinary Pathology to concentrate on pathologic anatomy rather than bacteriology, infectious disease, or toxicology.^42^ The institutionalisation of veterinary pathology also found favour among the seven new American veterinary colleges in America and with the Food and Drug Administration, keen to define the expertise necessary to evaluate and conduct animal testing regimes. Military and industrial research was entangled and Innes would continue pursuing government-funded research after leaving Brookhaven.^43^ Elsewhere in continental Europe, the Commonwealth, or Japan, the relationships of veterinary pathologists and the military differed, whether the field was already stronger or that funding opportunities varied. But even here American public health grants, other national government funding and supranational organisations such as the World Health Organisation were crucial in bolstering research opportunities and institutional settings for comparative neuropathology and veterinary pathology.^44^ Innes’s and Saunders’s career paths and the institutional configurations bolstering *Comparative Neuropathology* disclosed the continued, long-standing links between military institutions and veterinary medicine. It, moreover, revealed fears about a world of proliferating neuropathologies and a new post-war effort to describe, and exploit, the vulnerabilities of the nervous system.

## Acknowledging new neurological patients

Innes and Saunders’s 1957 chapter in *Advances in Veterinary Sciences* was a first English-language attempt to review the literature on “Diseases of the Central Nervous System of Domesticated Animals and Comparisons with Human Neuropathology.” Their publication overlapped with a German-language volume by two Swiss authors who aggregated their single-species handbooks on dogs, cows, and swine into a comprehensive account of comparative neuropathology.^45^ The publication of *Comparative Neuropathology* therefore signalled not only a world of proliferating neuropathological conditions but also changing markets for veterinary patients and their owners, including in veterinary neurology.^46^

Veterinarians, for instance, were turning to the literature to assess whether congenital malformations in Hereford cattle and other livestock were hereditary, associated with nutritional deficiencies or maternal ill-health, or due to environmental poisonings by plant or synthetic chemicals. Malformations had occurred before the 1950s, but changes in livestock production were altering morbidity rates and placing more emphasis on accurate diagnosis. Interbreeding caused by an increasing reliance on artificial insemination was amplifying degenerative hereditary conditions. Poisoning with herbicides, pesticides, and other newly synthesised substances were becoming more common. Questions of malnutrition had implications for the use of feeding supplements.^47^ Chapters on infectious diseases associated with bacteria, viruses, and parasites were, moreover, important for veterinary health officials faced with new outbreaks associated with livestock transported across continents.^48^ For veterinarians, whose practice concentrated on pets and small animals, rising disposable incomes and a new understanding of pets as “companion animals” were creating new consumer markets. By 1960 plans for preventative medical care, better animal hospitals, and a large range of veterinary pharmaceuticals offered new opportunities to see and treat animal patients for an expanded range of conditions and in well-resourced settings that paralleled those provided in human medical care. Knowledge of the nervous system, long considered to be too complex for non-specialists, was becoming obligatory for veterinarians who were assessing, and treating, cases of herniated disks, movement disorders, seizures, and non-infectious, chronic illnesses.^49^

New textbooks, published in the 1950s and 1960s, promised to make the field more accessible.^50^ To date veterinary neurology had been scattered among journal articles, reviews, and German-language textbooks. From 1956, English-speaking veterinarians treating canine patients could look to the handbook by John McGrath, a veterinary pathologist at the University of Pennsylvania’s veterinary school. *Neurologic Examination of the Dog with Clinicopathologic Observations* intended to translate expertise gained in neurophysiologists’ experiments on dogs into small animal practice.^51^ Introductory chapters sought to bridge experimental and clinical neurology, using experimental results of mid-brain resections, for instance, to explain clinical syndromes of exaggerated reflexes. Some reviewers suggested that such a structure was a more rational approach to clinical practice, yet McGrath’s second edition hewed more closely to clinical concerns.^52^ It increased the number of cases featured, discussed infectious diseases in a separate chapter, and elaborated on pathological conditions of the spinal cord, indicating the growing interest in a concise guide to neurological practice for veterinarians not based in research laboratories.

The later *Canine Neurology: Diagnosis and Treatment* (1965), written by a veterinary surgeon, presented an even larger world.^53^ It discussed electroencephalography, radiography, spinal fusions, and the medical management of traumatic brain injuries in detail, indicating that findings and techniques developed in human neurology were being adopted into veterinary practice to treat diseases in companion animals in similar detail as diseases in their human owners.^54^ The market was important, even though specialist centres acknowledged that these treatments constituted a small number of cases in general practice and study groups struggled to stay active.^55^ In 1961-62 the author of *Canine Neurology* had seen about 500 neurological cases, about a sixth of them surgical—less than 5% of the veterinary hospital’s total case load. Other estimates differed with some expecting one in ten veterinary cases to require neurological knowledge.^56^ Strikingly, about two thirds of the cases seen in Auburn were non-infectious, substantiating comparative neuropathologists’ claim that all mammalian nervous systems were subject to the same types of vulnerabilities, even if morbidity varied between species. It also illustrated the significant growth in the care for chronically ill pets and other animals.^57^

McGrath’s textbook showcased the overlap of forms of veterinary practices. His own account was based on a translation of practices in neurophysiological laboratories to clinical practice. It also found audiences in those caring for animals in toxicological laboratories. In his review of *Neurologic Examination of the Dog* Innes therefore commented,

Dogs are being used more and more by pharmacologists, toxicologists and pathologists for so-called chronic toxicity experiments. In some cases chemical compounds are given over many months to define possible toxic effects. Some ‘new drugs’ do produce neurologic signs often poorly defined; hence such workers concerned should firstly be aware of the variety of spontaneous neurologic conditions which affect dogs, and secondly they should know how to set out about studying such affected animals from both clinical and pathological angles.^58^

Elsewhere Innes had criticised “a tendency amongst some experimentalists to regard animals as test tubes, with an outside skin covering a variety of peculiar internal organs […]; the state of these organs is assumed to be immaterial, as long as the animal breathes and its heart throbs, so that it can be subjected to some experiment.”^59^ Even experiments that evaluated acute poisonings or ended in a quick death of the experimental animal could not ignore the possibility that spontaneously arising illnesses, clinically silent conditions, nutrition, or genetics affected experimental results. As Innes’s papers showed, even clinically silent conditions could have neuropathological implications. The previous section discussed that the inclusion of Minamata Disease, cerebellar malformations in Hereford cattle and nerve system damage due to pharmaceuticals highlighted that *Comparative Neuropathology* portrayed a world of proliferating neuropathologies, but the analysis of chronic diseases and growth of a literature dedicated to treating and assessing neurological conditions in animals also revealed a world of proliferating sites of comparative neurology and neuropathology which in turn were probing neuropathological conditions previously unknown or unexamined.

## Gathering data on neurologic disease

To document and organise the proliferating material on neurologic disease, Innes and Saunders leaned on classic techniques of histopathology to identify changes in nerve cells, lesions in nervous tissue, their distribution patterns and their possible correlation with clinical syndromes. They aimed to be “meticulous” in their histological analysis of lesions and “thoughtful,” possibly “imaginative,” in discussing causes, emphasising description of the diseases over the speculation of causes.^60^ Those interested in current virology or bacteriology were to look elsewhere. Instead, Innes and Saunders stressed the value of morphological analysis of nervous tissue, mainly by light microscopy and an array of simple stains, referring readers to the recently released *Greenfield’s Neuropathology* for its introductory chapters on neuropathology and to the last four decades of literature on the normal neuroanatomy of dogs, cats, sheep, goats, cattle, and horses to establish baselines. Their book collected cases from existing publications, personal experience, and correspondence networks, noting the perspectives of neurologists, veterinarians, veterinary pathologists, neuropathologists, and wet-bench laboratory researchers. The majority of diseases and disorders occurred spontaneously. Conditions were described variously by clinical features, laboratory tests, and histopathological findings with supplementary insights from heredity studies, toxicology, experimental studies of inoculation and transmission, epidemiological features and environmental conditions.

Earlier authors had resisted comparing neuropathological conditions across species, insisting that the lack of comprehensive knowledge about a species’ range of neurological disorders forbade accurate comparison.^61^ In contrast Innes and Saunders presented comparison as a more loosely structured empirical process. They sought to unify neurological disease descriptions internationally, firstly, to prove that neurologic diseases existed “on a global basis” and, secondly, to differentiate “the confusing field complexes [by recognising clear clinicopathological pictures].”^62^ Conceived as a snapshot of current knowledge, it was open to revisions as cases such as the barkers (a disorder in newborn foals), discussed among the miscellaneous diseases in 1957 and among the congenital conditions in 1962. Innes and Saunders’s approach mapped onto contemporaneous volumes that “freely changed perspective” throughout the book, considering neuropathology variously from the angle of “humans or human [neurological] illnesses; … specific animal species or [neurological] illnesses in animals; … classes of illnesses; … clinical practice; or particular organ systems” in order to locate overarching principles and correlate conditions in humans and animals.^63^ “Classification is not, however,” Innes and Saunders stressed, “an end to itself.”^64^

Particularly in contentious established categories, *Comparative Neuropathology* retraced the publication history and construction of disease classifications to illustrate historically contingent interpretations and undermine cherished neurological concepts. Innes and Saunders asked whether researchers had eagerly embraced demyelination as a definitive disease process due to use of myelin stains and due to academic interest in myelinating processes in early childhood.^65^ Its authors presented *Comparative Neuropathology* as a corrective for the many experimentalists, and presumably clinicians, who “[did] not read enough” to assess the novelty of their findings as Innes wrote elsewhere.^66^ It sought to align disease classifications across continents and veterinary traditions, showing that Peruvian *renguera* constituted the same entity as swayback pathologically and revealing cases of swayback in alpaca, llama, and other South American ruminants with similarly long gestation periods and patterns of foetal development.^67^ Understanding the global distribution of swayback, its geographically distinct features, and its resemblances to diseases in other species could therefore prompt new exploratory research into nerve growth, vulnerability to copper absorption, and other features of swayback.

Descriptions were bundled though the organisation was flexible. Early chapters outlined pressing specialist questions in research and practice including Fankhauser on assessing cerebrospinal fluid and the neurologist Ludo van Bogaert on primate neuropathology. A chapter on congenital and hereditary conditions was followed by chapters on infectious diseases, post-viral complications and experimental encephalitis, that occupied a third of the book. Subsequent chapters on pigmentation patterns sidled up to discussions on poisonings, tumours, spinal cord injuries, and a final chapter on “miscellaneous neurologic disorder” which “lumped” together conditions which neither “warranted” their own chapter nor “fit” existing categories.^68^ Gathering together disease entities and disorders, *Comparative Neuropathology* provided a snapshot of knowledge of neuropathological conditions in animals up to 1960 and emphasised the range of pathological conditions yet to be satisfactorily explained. It intended to show neuropathology as a thriving “intellectually challenging science” rather than simply a diagnostic tool or “a field full of pressing disease problems crying out for a solution [and control].”^69^

With the exception of Fankhauser’s chapter and some remarks on neurological examinations in primates, the volume sidelined questions about diagnostic methods in living animals, referring readers to McGrath’s *Neurologic Examination of the Dog* and other textbooks. “In effect,” Innes and Saunders wrote, “neurologic diagnosis in animals under ordinary circumstances is still primitive for tacit reasons, and in many cases it is not even tentatively clinical, but more etiologic, or based on clinical signs and ‘flock’ history.”^70^ Concentrating on diseases as problems and delineating treatment methods or diagnostic procedures, species by species, therefore struck Innes and Saunders as less fruitful and too limiting. Pathology was a science, they insisted, not merely a tool to confirm diagnoses post mortem. Nonetheless, the science of comparative neuropathology they envisaged was eminently rooted in clinical practice. Local particularities of the case mattered, rare cases and rare diseases were included in discussions, and the analysis hinged on the figure of the clinician in that it depended on clinical acumen in identifying typical pathological patterns in nervous tissue, differentiating normal and pathological changes, and noting clinical and pathological resemblances to diseases in other species. Comments indeed left little doubt of their personal opinions.

The case of scrapie demonstrated the potential of a more comparative approach. In 1959 one of Innes and Saunders’s co-authors, the pathologist William Hadlow (1921-2015), had written to the *Lancet* that the “enormous ballooned ‘holes’” in the brain tissue of sheep affected by scrapie resembled the pathological features observed in the brain tissue of humans affected by a rare degenerative disease, kuru.^71^ Though he hesitated to draw “too close an analogy between diseases of man and lower animals,” he expected” valuable clues [to] the fundamental nature.” “One might surmise that the pathogenetic mechanisms involved in scrapie—however unusual they may be—are unlikely to be unique in the province of animal pathology.”^72^ Others were soon highlighting similarities to Aleutian mink encephalopathy and hypothesising that this new class of diseases was caused by a “slow virus.”^73^ Conditions could now be classified as acute, chronic—or slow. Researchers subsequently commented on possible infectious agents, core resemblances, and transmission pathways between these diseases and Creutzfeld-Jakob disease. The case of scrapie vindicated those who considered neuropathology one of the most promising site for insights gained by analogy.^74^ However obscure a neurological disease or limited in the species it affected, diseases such as kuru, scrapie, and their ilk could model human neurological conditions or provide insights into fundamental research questions. The uncertainty “which human diseases [had] or [did not have] analogies in the domesticated animals,” therefore, amplified the potential reach of *Comparative Neuropathology*.^75^ As in the 1940s, the link to human neuropathology was crucial to the success of the field and its funding.

*Comparative Neuropathology* mapped onto high-profile research on neurological diseases. The chapter on primate neuropathology addressed recent difficulties in developing and testing polio vaccines, heavily reliant on primates and knowledge of primate neuropathology.^76^ Elsewhere large-scale vaccination drives were raising the profile of adverse neuropathological reactions to rabies treatment which needed to be identified, while iatrogenic damage to the nervous system due to antipsychotics and thalidomide were beginning to flood neuropathological research.^77^ Read in terms of professional reach, the “global basis” envisaged comparative neuropathology at the nexus of veterinarians in small animal practice, toxicology departments of private industry, military research into nerve agents, practices specialised in large animals or horses, bacteriological laboratories in public health campaigns, and academic departments of neurophysiology and pathology. The publisher, Academic Press, also advertised the volume to “geneticists, nutritionists, toxicologists, virologists, endocrinologists, embryologists” who might be interested in chapters on hereditary and congenital diseases, the effect of pellagra and other nutritional deficiencies on the brain, or the discussion of inflammatory conditions after viral infections or vaccination.^78^

Reception was mixed. A reviewer in the *Canadian Veterinary Journal* thought “the lengthy historical and clinical reviews … repetitive and wasteful of space,” wondering whether it was “reasonable to occupy some 28 column inches with clinical discussion of amaurotic epilepsy in primates … when only about four column inches are devoted to pathological description”—before concluding that the authors’ “personal opinions (subtly, in the guise of original material or otherwise)… add[ed] immeasurably to its value.”^79^ The *Archives of Neurology* praised it as “thought-provoking” for clinical neurologists, but expected pathologists to be its main audience, and the *Journal of Neuropathology and Experimental Neurology* thought it particularly valuable for experimentalists.^80^ For the reviewer in the *British Medical Journal*, Charles Lumsden, a British neuropathologist working on tissue culture, it “blazed a trail in [the] virgin forest” of a truly comparative pathology because it did not replicate species boundaries, but he bemoaned that the text failed to take account of human neuropathologists’ lack of knowledge about non-human mammalian nervous systems.^81^

In its collection of cases *Comparative Neuropathology* embodied a particular moment in the early 1960s when researchers, in probing the nervous system, also amassed an increasing body of knowledge about the vulnerabilities of the nervous system. To marshal these constituencies, Innes and Saunders, their co-authors, and colleagues gathered this information into a more coherent structure. In many ways, *Comparative Neuropathology* succeeded in demonstrating a “global basis for neurological disease.” It made neuropathological conditions in animals accessible and visible to scientific researchers and clinicians in cognate fields and amplified individual studies, such as those pursued by John McGrath in canine neurology, providing a further means of assessing the health of animals’ nervous systems whether in the field, farm, veterinary office, or laboratory. Studies of kuru and scrapie therefore had a platform of more scattered alternate sites. Conversely studies of these slow-virus diseases and the interest they generated in institutions dedicated to human disease drew a “rich uncle” to veterinary research.^82^ A decade later the editors of a newly founded journal of neuropathology praised “our veterinary colleagues” for their “great interest in the neurological diseases of domesticated animals that are not necessarily of great economic importance. This most welcome development is of especial value in the whole field of study [of neuropathology], bringing new ‘models’ of disease into our midst for future investigation.”^83^

This paper has shown that the veterinary pathologists’ work had a far larger reach. In the late 1960s Innes imagined a multi-authored volume with contributions from colleagues in North America and Europe, but *Comparative Neuropathology* was never revised or reissued.^84^ Instead, veterinary neuropathology was incorporated into new textbooks on veterinary neurology or specialist chapters in pathology handbooks which detailed central nervous system, peripheral nervous system, and eye.^85^ Nonetheless, *Comparative Neuropathology* did not constitute a blip, but mirrored other endeavours to consolidate rhetorical platforms and indicate common ground in medical and biological fields.

## Seeking common ground in the early 1960s: “One science” with “many and diverse techniques”

Innes and Saunders characterised themselves as “general pathologists interested in the brain as an organ” who refused to examine the brain without consideration of the body.^86^ Their stance clashed with the increasing focus on neuronal cells and their reactions, which defined contemporary biological research on the nervous system. *Comparative Neuropathology* benefited from renewed interest in human neuropathology, which was flourishing in the post-war period due to the expansion of neurosurgery, tissue cultures, and new techniques such as electron-microscopy and histochemical stains. Historically the field had been the purview of neurologists, psychiatrists, and neurosurgeons rather than pathologists, but Innes and Saunders’s incursion into neuropathology as veterinary pathologists coincided with a general re-alignment of neuropathology as an independent speciality within histopathology. Neuropathologists increasingly situated themselves as experts in identifying diseases of the nervous system and therefore vital team members for experimentalists, neurologists, and neurosurgeons. They argued that they alone were able to differentiate between spurious findings, coincidental pathologies, significant lesions, and resemblances to historical accounts, thereby guarding neurological sciences as a whole against the proliferation of disease classifications.^87^ The characterisation resonated with the well-worn trope of the clinical neurologist as well-educated generalist and masterful diagnostician and with other expansive definitions of the field which saw it unifying medicine, philosophy and its scion psychology, and the natural and biological sciences.^88^ Developed in the late nineteenth century, this imagery resurged among neurologists after the Second World War in an effort to stem specialisation, prompting organisers of the 1958 international congress of neurology to join separate conferences for electroencephalography, neurosurgery, clinical neurophysiology, neurology and neuropathology. Innes and Saunders were among the conference attendees whom speakers inveigled to share “one science” even if they used “many and diverse techniques.”^89^ In *Vergleichende Neuropathologie*, published a year earlier, Frauchiger and Fankhauser similarly suggested that comparative neuropathology was capable of providing this common ground.^90^

Innes and Saunders echoed this expansive definition, arguing that comparative neuropathology was “one of the broadest disciplines now in existence” which was “coming into its” as an “intellectually challenging science.”^91^ Institutional networks confirmed this narrative: Comparative neuropathology was represented in the new national and international societies for veterinary pathology.^92^ There were, moreover, dedicated working groups and reference laboratories at the World Federation of Neurology (WFN) and the International Brain Research Organisation (IBRO), both founded in the early 1960s.^93^ Contemporaneous research programmes centred on an interdisciplinary study of brain structure, learning, memory, consciousness, and information processing. They drew together researchers in biophysics, biochemistry, neuroanatomy, psychiatry, psychology and a flourishing cadre of cognate field. Mirroring the arrangements of inter-war comparative medicine, Innes and Saunders hoped to benefit from this funding, professional positions, and prestige associated with the neurosciences, the biological sciences, and medicine.

Their morphological approach, however, was at odds with contemporary research funded by organisations such as the National Science Foundation, which hardly supported anatomical studies.^94^ Innes and Saunders positioned themselves as important critical reviewers. Open to new neurohistological techniques they were scathing of those worshipping “pubescent” techniques to the detriment of integrating clinicopathological syndromes with histopathology and careful experimental design.^95^ Relying on histochemical stains, electron microscopy, or microbes alone was a wish to “escape into a brave new world,” according to Innes and Saunders.^96^ The vagaries of clinical cases and pathological anatomy featured in *Comparative Neuropathology* were to provide much needed depth to the dominant discourses of histochemistry and “microbe-hunting” or the quest for novel disease entities.^97^ Similar projects were underway in other biological sciences whether in setting up databanks to classify molecular data, standardise anatomical nomenclature, or systematise disease categories. For Innes, Saunders, and their peers, comparison guarded against over-specialisation and allowed researchers to trace the range of reactions to trauma, pathogens, environmental conditions, or evolutionary change as if to contextualise experimental results. Researchers, moreover, could consider a variety of sources—from historical case reports to bacteriological findings to epidemiological studies—to shape their discussions. Transcending limited sources and appropriating material across disciplinary boundaries was therefore encouraged, even as an insistence on meticulous histopathological analysis and clinical acumen set out to protect comparative neuropathology against incursions from other specialists.

In rebuffing an exclusively molecular focus *Comparative Neuropathology* chimed with organismic biology and similar efforts in the 1960s that rejected the molecularisation of biology and explanations of living processes in the terms of physical sciences. Carried by Ernst Mayr, organismic biology argued for a continued relevance of “Old,” naturalist Biology, its practices of collecting and comparing specimens, and its focus on environment and evolution.^98^ In contrast to the Mayr’ian biology—and nineteenth-century comparative neurology—, Innes and Saunders neglected evolutionary questions, although their collection of cases did elucidate the morphogenesis of the nervous system and its vulnerability to environmental conditions. Their insistence on species variety also concurred with similar comments in post-war comparative psychology and comparative physiology.^99^

Reflecting a heterogenous landscape in the neurological and the biological sciences *Comparative Neuropathology* sought to garner support from its multiple strands including the resurgence of comparative medicine. In his 1944 comments at the Royal Society of Medicine’s Section of Comparative Medicine, Innes had rejected any “real borderline between animal and human pathology,” suggesting than “such differences which do exist are more apparent than real.”^100^ Two decades later a new generation of advocates was realigning veterinary and (human) medicine. Veterinarians, veterinary public health officials, and veterinary pathologists argued that their insights into conditions in multiple species gave them a privileged vantage point to direct research and manage human and veterinary diseases and disorders.^101^ Rooted in cross-species comparison, these calls were a concerted push to transcend professional boundaries, access new resources and showcase the value of field-based practices and experimental work. Advocates, often veterinary public health professionals associated with large international health organisations, showcased zoonoses and infectious diseases, but some campaigners also discussed other causes of animal and human ill-health. By the late 1960s authors expanded the remit to chronic, degenerative, non-infectious illnesses such as those profiled in *Comparative Neuropathology*.^102^

Innes and Saunders did not have an immediate affiliation with the movement, but they and other comparative neuropathologists had close ties to the University of Pennsylvania (UPenn), where researchers had begun writing editorials gesturing towards “One Medicine” in the early 1960s—the term which would subsequently define the movement.^103^ As *Comparative Neuropathology* was published, UPenn’s veterinary school had just received a ten-year, million-dollar follow-on grant from the NIH to pursue comparative cardiology. Whereas the cardiovascular system was known to be a major cause of mortality in humans and was becoming the site of significant medical interventions, its pathologies and chronic conditions in animals were hardly understood. An earlier study, also at UPenn, had shown a surprising number of cases of congestive heart failure in canine patients. Similarities with human conditions justified “going to the dogs” as a new experimental model in medical studies, but findings had repercussions for veterinary and medical practice alike.^104^ The development paralleled discussions in canine nervous system—studied in John McGrath’s UPenn laboratory, and in Fankhauser’s research on brain angiopathies in pigs, pursued while a visiting fellow at UPenn in 1963. These reinforced the veterinary school’s concerted effort to bolster its standing in biological and medical research and compete for NIH grants.^105^ The strategy included affiliations with the pharmaceutical industry clustered around Philadelphia illustrated by Saunders’s roles as visiting and adjunct professor throughout his career at nearby Smith Kline French.

Innes and Saunders had positioned comparative neuropathology as an “intellectually challenging science [rather] than [as] a field full of pressing disease problems crying out for a solution so that they can be controlled,” distancing themselves from veterinary public health with its focus on preventing infectious disease transmission be it in animals or between animals and humans. Their work presented a world resolutely not limited to monitoring slaughterhouses, managing culling and vaccinating herds, hunting for infectious agents, or writing policy to control diseases, typically considered key in veterinary public health and later One Medicine. Yet Innes and Saunders’s relationships with researchers at UPenn also highlighted a second thread of One Medicine which still requires future elucidation. Historians have already noted One Medicine’s links to earlier comparative medicine traditions and the associated professional stakes. *Comparative Neuropathology*, however, also exposes contingencies yet underexplored in the making of One Medicine—alliances with pharmaceutical firms, use of military funding, and overlaps with the biological sciences.^106^ In assembling a global basis for assessing diseases, *Comparative Neuropathology* therefore touched upon multiple expanding fields beyond their immediate constituencies in neurology and veterinary medicine. It also disclosed veterinary and medical pathologists’ ongoing attempts to maintain their professional standing in medical research as biological studies became more prominent in the manifold investigations into the nervous system—and elsewhere.

## Conclusion

*Comparative Neuropathology*, firstly, disclosed the continued efforts to frame veterinary medicine as a biomedical subject, visible in Innes’s papers to the section of Comparative Medicine as well as Innes and Saunders’s plea for comparative neuropathology as more than disease prevention. In its range of cases *Comparative Neuropathology* therefore casts new light on efforts to bridge human and animal health, situating the One Medicine initiative in contemporaneous efforts to counterbalance medical specialisation and biological molecularisation. Innes and Saunders wrote *Comparative Neuropathology* while rooted in handling animals as experimental subjects, but the volume’s strength lay in attending to other circumstances of animal health and veterinary care, naturally occurring diseases and neuropathologies across species. Conceived as a corrective to exclusively laboratory-based studies of disease and its causes, it documented animals becoming neurological subjects in their own right after decades influencing neurology, neurophysiology, -anatomy, and cognate fields. The volume, moreover, indicated that this development relied on alliances with medical neurologists, pathologists, and other practitioners preoccupied with human health as well as on alliances in the diversifying field of veterinary medicine. *Comparative Neuropathology*’s support from the US armed forces, government research organisations, and pharmaceutical industry gestured towards additional new audiences for studies of neurotoxicity and other non-infectious conditions affecting nervous systems. These interfaces reinforce the analysis of the neurosciences as a multiply hybrid field, but also sketch out a rich landscape of biomedical research around 1960.

In attending to neuropathologies across multiple species *Comparative Neuropathology*, secondly, commented on an on-going discussion about species diversity in medical and biological research. Comparative medicine, as envisaged by Charnock Bradley and others in the 1920s, stressed the benefits of investigating spontaneous diseases in multiple species. These arguments had also underpinned Innes’s interwar papers which gestured towards uncovering the mechanism of demyelination while scrutinising lambs with swayback. Three decades later, *Comparative Neuropathology* remarked on swayback in alpaca, llama, and ruminants with similar foetal development to emphasise the value of considering diverse species in researching neuropathological conditions. These arguments have resurfaced in twenty-first century critiques of neuroscience and calls to balance studies of model organisms with investigations of biological diversity. A close reading of *Comparative Neuropathology* is a useful reminder of a longer trajectory of these remarks and the professional projects which underpin them.

## Funding

This work was supported by the Wellcome Trust (grant number: 092719/Z/10/A). I declare no conflict of interest.

## Footnotes

1 JRM Innes and LZ Saunders, *Comparative Neuropathology* (New York: Academic Press, 1962).

2 The anecdote—and Charcot’s “C’est un malade!”—are central to W.F. Bynum, “’C’est un malade’: Animal Models and Concepts of Human Diseases,” *J. Hist. Med. Allied Sci.* 3, no.1 (1990): 397-413. I am grateful to Abigail Woods for pointing out Charcot’s disciplinary affiliation.

3 For a longer history of this re-framing: Abigail Woods, “From Practical Men to Scientific Experts: British Veterinary Surgeons and the Development of Government Scientific Expertise, c1878-1919,” *Hist. Sci.* 51, no. 4 (2013): 457-80.

4 See for instance, Gordon M. Shepherd, *Creating Modern Neuroscience: The Revolutionary 1950s* (Oxford: Oxford University Press, 2009), 5 which announces that “the history of neuroscience extends across all species,” continuing “experiments on a wide range of animal species are crucial,” thereby conflating animals with experimental animals.

5 Joelle M. Abi-Rached and Nikolas Rose, “The Birth of the Neuromolecular Gaze,” *Hist. Hum. Sci.*, 23, no.1 (2010), 11-36.

6 Nikolas S Rose and Joelle M. Abi-Rached, *Neuro: The New Brain Sciences and the Management of the Mind* (Princeton, NJ: Princeton University Press, 2013), chapter 3, “What’s Wrong With Their Mice?,” 109. For a telling view “from the animal”: Fabio De Sio, “Leviathan and the Soft Animal: Medical Humanism and the Invertebrate Models for Higher Nervous Functions, 1950s–90s,” *Med. Hist.*, 55, no.3 (2011): 369-74. De Sio’s analysis disrupts the human brain-centric historiography of neuroscientific research, which pervades Rose and Abi-Rached’s account. This paper too leans on the “animal turn” which has unearthed animals to be instrumental in constructing medical knowledge and professional identities: Abigail Woods, Michael Bresalier, Angela Cassidy, and Rachel Mason Dentinger, *Animals and the Shaping of Modern Medicine: One Health and its Histories* (Palgrave-Macmillan, 2018): 247-68.

7 Abi-Rached and Rose, “Birth,” 12.

8 ST Casper and D Gavrus, *The History of Brain and Mind Science: Technique, Technology, Therapy* (Rochester, NY: University of Rochester Press, 2017), see also the special issue on limits of method in the neurosciences, particularly those aligned with neurology, *Can. Bull. Med Hist.*, 33, no. 2 (2016) and M Stadler, “The Neuromance of Cerebral History” in S Choudhury and J Slaby, eds, *Critical Neuroscience: A Handbook of the Social and Cultural Contexts of Neuroscience* (John Wiley & Sons, 2012), 135-58, which troubles dominant human-brain centric accounts.

9 Warwick Anderson, “The Possession of Kuru: Medical Science and Biocolonial Exchange,” *Comp. Stud. Soc. Hist.*, 42, no.4 (2000): 713-44; *The Collectors of Lost Souls: Turning Kuru Scientists into Whitemen* (Baltimore: Johns Hopkins University Press, 2008); Kiheung Kim, *Social Construction of Disease: From Scrapie to Prion* (London: Routledge, 2006). Rachel Mason Dentinger and Abigail Woods, eds, “[Topical Collection:] Working Across Species: Comparative Practices in Modern Medical, Biological and Behavioural Sciences,” *Hist. Phil. Life Sci.* 40 (2018), not limited to disciplines aligned with natural history: Bruno J. Strasser and Soraya de Chadarevian, “The Comparative and the Exemplary: Revisiting the Early History of Molecular Biology,” *Hist Sci.* 49, no. 3 (2011): 317-36.

10 Stephen Casper, “The Collectors of Lost Souls: Turning Kuru Scientists into Whitemen by Warwick Anderson The Social Construction of Disease: From Scrapie to Prion by Kiheung Kim,” *J. Hist. Neurosci.* 20 (2011): 160-66 notes lacunae surrounding neurologists’ entwinement with military institutions and the pharmaceutical industry, far better documented in psychiatry.

11 Abigail Woods and Michael Bresalier, “One Health, Many Histories,” *Vet. Rec.*, 174, no. 26 (2014): 650-54. The term, One Medicine, is commonly associated with Calvin Schwabe’s 1984 edition of *Veterinary Medicine and Human Health* (Baltimore, Md: Williams and Wilkins), but authors affiliated with the University of Pennsylvania used the term in medical publications from the early 1960s: Angela Cassidy “Humans, Other Animals, and ‘One Health’ in the Early Twentieth-First Century,” in Woods *et al.*, *Animals*: 193-236, 200-205.

12 Abigail Woods, “Animals and Disease,” in *Routledge History of Disease*, ed. Mark Jackson (London: Routledge, 2016), 147-64; Anne Hardy, “Animals, Disease and Man: Making Connections,” *Perspect. Biol. Med.* 46 (2003): 200-215.

13 Hermann Dexler, “Die Erkrankungen des Zentralnervensystems der Tiere” in: Albrecht Bethe et al. *Handbuch der normalen und pathologischen Physiologie, vol X: Spezielle Physiologie des Zentralnervensystems der Wirbeltiere* (Berlin: Springer, 1927), 1232-68.

14 JRM Innes, “[Discussion on the Limitations and Uses of the Comparative Method in Medicine],” *Proc. R. Soc. Med*. (1944): 653-55, 653.

15 See the long-established *Journal of Comparative Neurology*, founded in 1891, focused on comparative neuroanatomy in a zoological tradition.

16 L. Wilkinson, *Animals and Disease: An Introduction to the History of Comparative Medicine* (Cambridge: Cambridge University Press, 1992). Woods and Bresalier, “One Health,” 651-53.

17 O. Charnock Bradley, “What is Comparative Medicine?,” *Proc. R. Soc. Med.* 21 (1927), 129-34. Michael Bresalier, Angela Cassidy, and Abigail Woods “One Health in History,” *One Health: The Theory and Practice of Integrated Health Approaches* (Oxfordshire: CABI, 2015): 1-15, 7-8.

18 On veterinary research in early-twentieth-century Britain: Abigail Woods, *A Manufactured Plague? The History of Foot and Mouth Disease in Britain* (London: Earthscan, 2004), “The Science: 1912-1958”; *idem*, “From Coordinated Campaigns to Watertight Compartments: Diseased Sheep and their Investigation in Britain, c1880-1920,” in: Woods et al, *Animals*: 71-117.

19 Including a medical fellowship from the Rockefeller Foundation to spend two years in German institutes of pathology and a collaboration with medical pathologists in Edinburgh: W.F. Harvey, E.K. Dawson, and J.R.M. Innes, *Debatable Tumours in Human and Animal Pathology* (Edinburgh: Oliver and Boyd, 1940). E. Cotchin, “Obituary Notice: James Robert Maitland Innes: 25 October 1903-21 December 1974,” *J. Path.*, 119 (1976): 187-91. Mearl F. Staunton, “James Robert Maitland Innes,” *Bull. Soc. Pharmacol. Environ. Pathol.* 2, no. 1 (1974): 11-15, LZ Saunders, *A Biographical History of Veterinary Pathology* (Lawrence, KS: Allen Press, 1996), 244-45.

20 JRM Innes, “[Discussion],” 655; *idem*, “[Discussion on Nervous Affections in Man and Animals,” *Proc. R. Soc. Med.* (1940), 169-72.

21 JRM Innes, “The Pathology of ‘Swayback’—A Congenital Demyelinating Diseases of Lambs with Affinities to Schilder’s Encephalitis,” *Rep. Inst. Animal Pathol. Camb. Univ.* 4 (1936), 227-50.

22
*Ibid.*, 228.

23 J.R.M Innes, “Swayback: A Demyelinating Disease of Lambs with Affinities to Schilder's Encephalitis and Its Prevention by Copper,” *J. Neurol. Psychiatry*, 2, no. 4 (1939): 323-34, 323; Innes’ papers on swayback also appeared in *Vet. Rec.*, 48 (1936): 1539-49, *Proc. R. Soc. Med.* (1936): 406-409; and with G.D. Shearer in *J. Comp. Pathol. Ther.* 53, no. 1 (1940), 1-41.

24 A.M.G. Campbell, P. Daniel, R.J. Porter, W. Ritchie Russell, Honor V. Smith and J.R.M. Innes, “Disease of the Nervous System Occurring among Research Workers on Swayback in Lambs,” *Brain*, 70, no. 1 (1947): 50-58.

25 HJ Scherer, *Die Pathologie des Nervensystems der Säugetiere, unter besonderer Berücksichtigung der Primaten, ein Versuch* (Leipzig: G. Thieme, 1944). [*The Pathology of the Mammalian Nervous System with Particular Consideration of Primates, an Attempt)*, 255. E Frauchiger and R Fankhauser, *Vergleichende Neuropathologie der Menschen und Tiere* (Berlin: Springer, 1957), 96. JG Greenfield, *Neuropathology* (London: Arnold, 1958), 286-87.

26 Innes and Saunders, *Comparative Neuropathology*, 577.

27
*Ibid.*, xi, 38.

28
*Ibid*, 2-3.

29
*Ibid*, 802ff. Timothy S. George, *Minamata: Pollution and the Struggle for Democracy in Postwar Japan* (Cambridge, Massachusetts: Harvard University Press, 2001).

30 JRM Innes and LZ Saunders, “Diseases of the Central Nervous System of Domesticated Animals and Comparisons with Human Neuropathology” in *Advances in Veterinary Science*, vol 3, ed. CA Brandly and EL Jungherr (New York: Academic Press, 1957), 33-196, 147-48.

31 In the inter-war period, while still at Cambridge, Innes had pursued some experimental research on the effects of nutritional deficiencies or large doses of vitamins on the nervous system and other organs.

32 JRM Innes, “Veterinary Pathology: Retrospect and Prospect,” *Vet. Rec. 85* (1969): 730-41, 737 and 731. Military institutions had, of course, sustained veterinarians’ efforts to professionalise since the establishment of the first veterinary schools in the 1760s.

33 Staunton, “Innes,” 13.

34 Imperial Chemical Industries Limited: Pharmaceuticals Division, *Pharmaceutical Research in I.C.I, 1936-57* (Birmingham: Kynoch Press, c1958).

35 Innes and Saunders, *Comparative Neuropathology*, xiii-xiv. David C Dodd, “In Memoriam: Leon Z Saunders,” *Vet. Pathol.*, 46 (2009): 561–62; FF Katz, “Leon Saunders, William Osler and Rudolf Virchow: Their Contributions to Veterinary Pathology and ‘One Medicine’,” *Vet. Herit.* 37, no.1 (2014): 3-10. Saunders had come to Edgewood a year earlier than Innes, but left the year Innes arrived. Their collaboration dated to 1954, when Innes was still at Edgewood and Saunders at Brookhaven where he had moved in 1952.

36 Both Innes and Saunders moved into private industry subsequently: Saunders to Smith Kline French in Philadelphia, Innes to smaller laboratories in Washington DC.

37 As well as biological warfare agents. Ulf Schmidt, *Secret Science: A Century of Poison Warfare and Human Experiments* (Oxford: Oxford University Press, 2015); Edward Russell, *War and Nature: Fighting Humans and Insects with Chemicals from World War I to Silent Spring* (Cambridge: Cambridge University Press, 2001); Rob Evans, *Gassed: British Chemical Warfare Experiments on Humans at Porton Down* (London: House of Stratus, 2000). On the involvement of neurological scientists in chemical weapons research: Florian Schmaltz, “Neurosciences and Research on Chemical Weapons of Mass Destruction in Nazi Germany,” *J. Hist. Neurosci.*, 15, no. 3 (2006): 186-209.

38 National Archives, College Park, Maryland: RG175, Box 62: Records of the Chemical Warfare Service: General Correspondence, Station Series, 1955-1959, declassified, “Army Chemical Center, Maryland: Chemical warfare laboratories: personnel inventory and appraisal form,” 19 October 1956. Innes is also listed as “Chief of Pathology Branch, Medical Laboratories, Army Chemical Center, Edgewood” in *Comparative Neuropathology.*

39 Schmidt, *Secret Science*, 213.

40 J.R.M. Innes, A. J. McAdams, and P. Yevich, “Pulmonary Disease in Rats: A Survey with Comments on “Chronic Murine Pneumonia,” *Am. J. Pathol.*, 32, no. 1 (1956): 141-59; J.R.M. Innes, “The Need for Critical Evaluation of Choice of Animal Species for Continuous Inhalation Exposure Experiments” in *Aerospace Medical Research Laboratories, Proceedings of the Conference on Atmospheric Contamination in Confined Spaces 30 March – 1 April 1965* (1965), 34-45; J.R.M. Innes and A. Carsten, “Demyelinating or Malacic Myelopathy: A Delayed Effect of Localized X-Irradiation in Experimental Rats,” *Arch. Neurol.*, 4, no. 2 (1961): 190-99.

41 Russell, *War and Nature*.

42 Innes was a Charter Member of ACVP, Saunders one of the first to pass the qualifying exams and later the editor of the *Pathologia Veterinaria* founded in 1964. TC Jones, “Early History of the American College of Veterinary Pathologists: 1947-1960,” *Vet. Pathol.* 27 (1990): 468-520; NF Cheville, “The Origins, Founding, and Early Development of the Journal *Veterinary Pathology*,” *Vet. Pathol.* 50 (2013): 939-45.

43 See the inclusion of Agent Orange components on a study of pesticides, insecticides, herbicides and other chemical compounds for the American National Cancer Institute: J.R.M. Innes *et al*, “Bioassay of Pesticides and Industrial Chemicals for Tumorigenicity in Mice: A Preliminary Note,” *J. Natl Cancer Inst.*, 42 (1969): 1101-14.

44 See the funding from the Swiss National Fund, the National Institute of Health, and US Public Health funds for the institute of comparative neuropathology at the veterinary school in Bern, Switzerland, where Fankhauser, one of Innes and Saunders’ co-authors, was based. Saunders had provided support during the application process: Fankhauser, letter to W. Hofmann, dean, veterinary school, Bern, 28 January 1959, Folder 4551, Fankhauser papers, Archives of the Schweizerischen Vereinigung für Geschichte der Veterinärmedizin, Basel [“Fankhauser papers” in the following]. On other funding sources, see also description in “Führer durch die veterinär-medizinische Fakultät der Universität Bern,“1966 (brochure), folder, “Baugeschichte Fak. Bern,” uncatalogued archives, library, Vet Suisse, Bern.

45 The neurologist Ernst Frauchiger (1903-1975) and the veterinarian Rudolf Fankhauser (1919-1995), based in Bern, Switzerland (see footnote 44). Frauchiger’s interest in comparative neuropathology was steeped in interwar holism. For a programmatic statement see E Frauchiger, “Vergleichende Neurologie,” *Nervenarzt*, 34, no. 3 (1963): 105-107.

46 For histories of animal patients, though not neurological patients, particularly in the context of veterinary surgery: Chris Degeling, “Negotiating Value: Comparing Human and Animal Fracture Care in Industrial Societies,” *Sci. Tech. Hum. Values*, 34, no. 1 (2009): 77-101; Thomas Schlich, Eric Mykhalovskiy, and Melanie Rock, “Animals in Surgery—Surgery in Animals: Nature and Culture in Animal-human Relationship and Modern Surgery,” *Hist. Phil. Life Sci.*, 31 (2009): 321-54; Susan D. Jones, *Valuing Animals: Veterinarians and Their Patients in Modern America* (Baltimore: Johns Hopkins Press, 2003). *The Neurological Patient in History*, ed. L. Stephen Jacyna and Stephen T. Casper (Rochester, NY: University of Rochester Press, 2012), otherwise expansive, only discusses human patients.

47 L.Z. Saunders, “Some Hereditary Neurologic Diseases in Animals,” in *Symposion über vergleichende Neuropathologie*, ed. E. Frauchiger and F. Seitelberg (Berlin: Springer, 1962), 52-53.

48 See for example the case of scrapie, discussed below.

49 Jones, *Valuing Animals*, chapter, “Pricing the Priceless Pet.”

50 An early German-language example: Ernst Frauchiger and Rudolf Fankhauser, *Die Nervenkrankheiten unserer Hunde* (Bern: Hans Huber, 1949), considered for translation into English in 1959: Fankhauser—W.Koella correspondence, 16 January 1959 and 13 August 1959, Folder 4551, Fankhauser papers.

51 John McGrath, *Neurologic Examination of the Dog with Clinicopathologic Observations* (London: Henry Kimpton, 1956), 9. A second edition was published in 1960. See also: AC Palmer, *Introduction to Animal Neurology* (Oxford: Blackwell, 1965).

52 Neal Nathanson, “Neurologic Examination of the Dog with Clinicopathologic Observations. John T. McGrath,” *Quart. Rev. Biol.* 34 (1959): 170.

53 McGrath, *Neurologic Examination*, 20

54 BF Hoerlein, *Canine Neurology: Diagnosis and Treatment* (Philadelphia: WB Saunders, 1965).

55 AC Palmer, “First Attempt to form the BSAVA Neurological Study Group in 1968,” *Vet. Hist.*, 14 (2009): 349-53.

56
*Veterinärmedizinische Neurologie: Ein Leitfaden für Studium und Praxis*, ed. Marc Vandevelde, Andre Jaggy and Johann Lang, 2nd rev. ed (Berlin: Parey Buchverlag, 2001 [1986]). Frauchiger and Fankhauser, *Vergleichende Neuropathologie* estimated that 3-8% veterinary cases were related to the nervous system, though they expected a higher, hidden number.

57 On the co-constitution of new diseases and their treatment by ill pets, their owners, and veterinarians in this period: Susan Jones, “Framing Animal Disease: Housecats with Feline Urological Syndrome, Their Owners, and Their Doctors,” *J. Hist. Med. Allied Sci.*, 52 (1997): 202-35.

58 J.R.M. Innes, “[Review:] Neurologic Examination of the Dog with Clinicopathologic Observations,” *J. Neuropathol. Exp. Neurol.*, 19, no. 4 (1960): 630-31.

59 J.R.M. Innes, “The Nature and Variety of Diseases of Laboratory Animals and their Significance to Medical Research,” *N. Y. Pathol. Soc.*, 33, no. 11 (1957): 807-810; 807. For Innes’ papers on this subject: J.R.M. Innes, E. J. Donati, and Paul P. Yevich, “Pulmonary Lesions in Mice Due to Fragments of Hair, Epidermis and Extraneous Matter Accidentally Injected in Toxicity Experiments,” *Am. J. Pathol.*, 34, no. 1 (1958): 161-67; J.R.M. Innes *et al*, “Occult Endemic Encephalitozoönosis of the Central Nervous System of Mice,” in *Symposion über vergleichende Neuropathologie*, ed. E. Frauchiger and F. Seitelberger (Berlin: Springer, 1962), 97-99.

60 Praise for a peer in Innes and Saunders, *Comparative Neuropathology*, 422, in a footnote.

61 Scherer, *Pathologie des Nervensystems.*

62 Innes and Saunders, “Diseases of the Central Nervous System,” 83.

63 Frauchiger and Fankhauser, *Vergleichende Neuropathologie*, 32.

64 Innes and Saunders, *Comparative Neuropathology*, 1.

65
*Ibid*, chapter XIV on demyelinating diseases. On the value of retracing the construction of disease categories and disciplinary customs: Innes, “Veterinary Pathology,” 732.

66 J.R.M. Innes, “People Do Not Read Enough or Very Wisely,” *J. Natl Cancer Inst.*, 35, no. 1 (1965): vi-vii.

67 Innes and Saunders, *Comparative Neuropathology*, 589.

68
*Ibid*, 799.

69
*Ibid*, xi.

70
*Ibid*, 3.

71 Anderson, *Collectors*; Kim, *Social Construction of Disease*.

72 W.J. Hadlow, “[Letters to the Editors:] Scrapie and Kuru,” *Lancet*, 274, no. 7097 (1959): 289-90.

73 Subsequently identified as prions.

74 WIB Beveridge, *Frontiers in Comparative Medicine* (Minneapolis: University of Minnesota Press, 1972). Kim, *Social Construction of Disease*, particularly 102-105. The identification of bovine spongiform encephalopathy (BSE) and its link to variant Creutzfeld-Jakob disease further bolstered this argument.

75 Innes and Saunders, *Comparative Neuropathology*, 3.

76
*Ibid*, 56. Anita Guerrini, *Experimenting with Humans and Animals: from Galen to Animal Rights* (Baltimore: Johns Hopkins University Press, 2003), chapter 6, “Polio and Primates,” particularly 114-29.

77 J.R.M. Innes, “Experimental ‘Allergic’ Encephalitis: Attempts to Produce the Disease in Sheep and Goats,” *J. Comp. Pathol. Ther.*, 61 (1951): 241-52, cited in: George A. Jervis, “Experimental Allergic Encephalitis in Animals, and Its Bearing upon the Etiology of Neuroparalytic Accidents Following Antirabies Treatment in Man,” *Bull. World Health Organ.*, 10, no. 5 (1954): 837-44. For an instance of the increasing visibility of iatrogenic neuropathologies, see the programme of the international congress of neuropathologists in Zurich in 1965.

78 Promotional material accompanying letter from Lore Henlein, Academic Press, to R. Fankhauser, 9 June 1960, folder 4552, Fankhauser papers. Academic Press also published *Advances in Veterinary Science* and *International Review of Neurobiology*, both advertised on the flyer.

79 T.J. Hulland, “Book Review: *Comparative Neuropathology* […],” *Can. Vet. J.*, 3, no. 12 (1962): 388.

80 Abner Wolf, “Comparative Neuropathology,” *J. Neuropathol. Exp. Neurol.*, 23, no. 2 (1964*):* 391; Richard Doyle, “Advances in Veterinary Science, “*AMA Arch. Neurol.*, 1, no. 6 (1959): 703-704, respectively.

81 CE Lumsden, “Comparative Neuropathology,” *Brit. Med. J.*, 1, no. 5347 (1963): 1730. For (veterinary) pathologists, knowledge of human diseases was a given: Innes and Saunders, *Comparative Neuropathology*, xi.

82 RF Marsh, “Slow Virus Diseases of the Central Nervous System,” *Advances in Veterinary Sciences* 18 (1974): 155-78.

83 “Editorial Preface,” *Neuropathol. Appl. Neurobiol.*, 1 (1975): v-vi.

84 Including Luginbühl, Fankhauser, Hadlow, and McGrath. Saunders “wanted nothing to do with this”: JRM Innes to R. Fankhauser, 6 June 1968, folder 4555, Fankhauser papers.

85
*[Joest’s] Handbuch der speziellen Anatomie der Haustiere*, 3rd ed, vol 3, ed. Johannes Dobberstein *et al* (Berlin: P. Parey, 1968), for instance, included a chapter by Saunders on the eye and by Fankhauser and Hansrüdi Luginbühl on the central and peripheral nervous system; see also L Saunders and LF Rubin, *Ophthalmic Pathology of Animals: An Atlas and Reference Book* (Basel: Karger, 1975).

86 Innes and Saunders, *Comparative Neuropathology*, xi and 500, respectively.

87 Schoefert, Anna Kathryn. “Neither Physicians Nor Surgeons: Whither Neuropathological Skill in Post-war England?,” *Medical History* 59, no. 3 (2015): 404-420.

88 Stephen J. Casper, “History and Neuroscience: An Integrative Legacy,” *Isis*, 105, no. 1 (2014): 123-32, *idem*, *The Neurologists: A History of a Medical Specialty in Modern Britain, c. 1789–2000* (Oxford: Oxford University Press, 2014), particularly “The Integrative Legacy of Contemporary Specialists in Neurology,” 153-78; *idem* and Rick Welsh, “British Romantic Generalism in the Age of Specialism, 1870-1990,” *Soc. Hist. Med.*, 29, no. 1 (2016): 154-74.

89 Francis Walshe, “Neurology at the Crossroads,” *Neurol.*, 8, no. 10 (1958): 796-98, 796.

90 Frauchiger and Fankhauser, *Vergleichende Neuropathologie*, 4.

91 Innes and Saunders, *Comparative Neuropathology*, preface xii. Innes, “People.”

92 In the US (1948), Germany (1952), USSR (1959), and as a world federation (1963) and as a specialist journal, *Pathologia Veterinaria* (1963): Saunders, *Biographical History*, 5.

93 Frauchiger and Fankhauser’s collection of tissue samples in Berne, for instance, was a WFN reference collection: Innes and Saunders, *Comparative Neuropathology*, 420.

94 Toby Appel, *Shaping Biology: The National Science Foundation and American Biological Research, 1945-1975* (Baltimore: The Johns Hopkins University Press, 2000), 72.

95 Innes and Saunders, *Comparative Neuropathology*, 689-90.

96
*Ibid*, 690.

97 J.R.M. Innes to R. Fankhauser, 20 June 1959, Folder 4551, Fankhauser papers.

98 Erika Lorraine Milam, “The Equally Wonderful Field: Ernst Mayr and Organismic Biology,” *Hist. Stud. Nat. Sci.*, 40, no. 3 (2010): 279-317.

99 Dentinger Woods, eds, “Working Across Species.”

100 Innes, JRM Innes, “[Discussion on the Limitations].”

101 For example, Calvin Schwabe, *Veterinary Medicine and Public Health* (London: Baillière Tindall & Cassell, 1964), second edition in 1969.

102 Woods, Bresalier, Cassidy, “One Health in History.”

103 Cassidy, “Humans, Other Animals and ‘One Health’,” 200-205. Carl F. Schmidt, “[Editorial:] One Medicine for More Than One Word,” *Circ. Res.*, 11 (1962): 901-903. Innes and Saunders’ Swiss co-author, Fankhauser, spent several months in McGrath’s laboratory, his student, Hans-Ruedi Luginbühl, several years: Saunders, *Biographical History*, 419.

104 H.L. Conn, D.K. Detweiler, J. McGiff and R.J. Luchi, “Why We Have Gone to the Dogs,” *Trans. Am. Clin. Climatol. Assoc.*, 77 (1966): 34-47.

105 In 1951 the school supported three projects worth about $24,000; ten years later 51 projects worth about $540,000: John E Martin, *A Legacy and a Promise: The First One Hundred Years, 1884-1984, School of Veterinary Medicine, University of Pennsylvania* (Philadelphia: Trustees of the University of Pennsylvania, 1984), 101-105. Joan Hendricks, Charles D Newton and Arthur Rubinstein, “’One Medicine – One Health’ at the School of Veterinary Medicine of the University of Pennsylvania – The First 125 Years,” *Vet. ital.*, 45, no. 1 (2001): 183-94.

106 On One Medicine, and its subsequent iterations as One Health, as a “response mounted by specific researchers (and policymakers) to problems manifesting at particular times and in particular places”: Cassidy, “Humans, Other Animals and ‘One Health’,” 196.

